# Fumonisin and Beauvericin Chemotypes and Genotypes of the Sister Species *Fusarium subglutinans* and *Fusarium temperatum*

**DOI:** 10.1128/AEM.00133-20

**Published:** 2020-06-17

**Authors:** M. Veronica Fumero, Alessandra Villani, Antonia Susca, Miriam Haidukowski, Maria T. Cimmarusti, Christopher Toomajian, John F. Leslie, Sofia N. Chulze, Antonio Moretti

**Affiliations:** a Research Institute on Mycology and Mycotoxicology, National Research Council of Argentina, National University of Rio Cuarto, Rio Cuarto, Cordoba, Argentina; b Institute of Sciences of Food Production, CNR, Bari, Italy; c Department of Plant Pathology, Kansas State University, Manhattan, Kansas, USA; Nanjing Agricultural University

**Keywords:** comparative genomics, gene inactivation, maize pathogens, mycotoxin biosynthesis, secondary metabolism, Argentina, *Fusarium subglutinans*, *Fusarium temperatum*, UPLC-MS, beauvericin, fumonisins, genome sequence, mycotoxins, plant pathogens

## Abstract

Fusarium subglutinans and Fusarium temperatum are common maize pathogens that produce mycotoxins and cause plant disease. The ability of these species to produce beauvericin and fumonisin mycotoxins is not settled, as reports of toxin production are not concordant. Our objective was to clarify this situation by determining both the chemotypes and genotypes for strains from both species. We analyzed 25 strains from Argentina, 13 F. subglutinans and 12 F. temperatum strains, for toxin production by ultraperformance liquid chromatography mass spectrometry (UPLC-MS). We used new genome sequences from two strains of F. subglutinans and one strain of F. temperatum, plus genomes of other *Fusarium* species, to determine the presence of functional gene clusters for the synthesis of these toxins. None of the strains examined from either species produced fumonisins. These strains also lack *Fum* biosynthetic genes but retain homologs of some genes that flank the *Fum* cluster in Fusarium verticillioides. None of the F. subglutinans strains we examined produced beauvericin although 9 of 12 *F. temperatum* strains did. A complete beauvericin (*Bea*) gene cluster was present in all three new genome sequences. The *Bea1* gene was presumably functional in F. temperatum but was not functional in F. subglutinans due to a large insertion and multiple mutations that resulted in premature stop codons. The accumulation of only a few mutations expected to disrupt *Bea1* suggests that the process of its inactivation is relatively recent. Thus, none of the strains of F. subglutinans or F. temperatum we examined produce fumonisins, and the strains of F. subglutinans examined also cannot produce beauvericin. Variation in the ability of strains of F. temperatum to produce beauvericin requires further study and could reflect the recent shared ancestry of these two species.

**IMPORTANCE** Fusarium subglutinans and F. temperatum are sister species and maize pathogens commonly isolated worldwide that can produce several mycotoxins and cause seedling disease, stalk rot, and ear rot. The ability of these species to produce beauvericin and fumonisin mycotoxins is not settled, as reports of toxin production are not concordant at the species level. Our results are consistent with previous reports that strains of F. subglutinans produce neither fumonisins nor beauvericin. The status of toxin production by F. temperatum needs further work. Our strains of F. temperatum did not produce fumonisins, while some strains produced beauvericin and others did not. These results enable more accurate risk assessments of potential mycotoxin contamination if strains of these species are present. The nature of the genetic inactivation of BEA1 is consistent with its relatively recent occurrence and the close phylogenetic relationship of the two sister species.

## Abstract (precis)

Fusarium subglutinans and F. temperatum are sister species and maize pathogens commonly isolated worldwide that can produce several mycotoxins and cause seedling disease, stalk rot, and ear rot. The ability of these species to produce beauvericin and fumonisin mycotoxins is not settled, as reports of toxin production are not concordant at the species level. Our results are consistent with previous reports that strains of F. subglutinans produce neither fumonisins nor beauvericin. The status of toxin production by F. temperatum needs further work. Our strains of F. temperatum did not produce fumonisins, while some strains produced beauvericin and others did not. These results enable more accurate risk assessments of potential mycotoxin contamination if strains of these species are present. The nature of the genetic inactivation of BEA1 is consistent with its relatively recent occurrence and the close phylogenetic relationship of the two sister species.

## INTRODUCTION

Fusarium subglutinans is an important pathogen of maize commonly isolated worldwide and is considered a causal agent of seedling disease, stalk rot, and ear rot (1). This species also can produce a broad range of mycotoxins (2). Within the morphological F. subglutinans *sensu lato* species, two populations were identified based on DNA sequence data (3). The two populations, F. subglutinans group 1 and F. subglutinans group 2, appeared to be reproductively isolated in nature and were presumed to be in the process of sympatric genetic divergence (3). Fusarium subglutinans group 1 has now been formally described as Fusarium temperatum (4), while F. subglutinans group 2 has retained the formal Fusarium subglutinans *sensu stricto* name.

Mycotoxin production by these species is of particular interest because production of beauvericin, a cyclic hexadepsipeptide with insecticidal and carcinogenic properties (5–7), has been reliably reported only in F. temperatum (group 1) and not in F. subglutinans (8–11). Beauvericin production has been used to identify the species to which some strains belong (11). Continuing studies of F. temperatum and F. subglutinans on cereals, primarily maize (12–20), have resulted in a general consensus that beauvericin is produced only by strains of F. temperatum and not by strains of F. subglutinans, but the genetics underlying these differences has not been investigated in any detail. Differences in beauvericin production by these two closely related species could provide insights into the evolutionary processes involved in their separation into different species.

The beauvericin (*Bea*) biosynthetic gene cluster was first described in Fusarium fujikuroi IMI 58289 and consists of a four-gene cluster: *Bea1*, which encodes the NRPS22, the nonribosomal peptide synthase responsible for synthesizing the beauvericin backbone, and *Bea2*, *Bea3*, and *Bea4*, which encode proteins with transport and regulatory functions (21). Orthologous four-gene biosynthetic clusters also are known in Fusarium proliferatum, Fusarium mangiferae, and Fusarium oxysporum (21), all of which are reported as beauvericin producers in multiple studies (22, 23). F. proliferatum is a common contaminant of cereals such as maize, wheat, and barley and can contaminate these substrates with beauvericin as well (22). F. mangiferae is a major cause of mango malformation worldwide (24), but a role for beauvericin in its phytotoxicity has not yet been identified. In F. oxysporum, a causal agent of tomato wilt, beauvericin reduces the level of ascorbic acid in the tomato cells, leading to the collapse of the ascorbate system and protoplast death (25).

The fumonisin (*Fum*) biosynthetic gene cluster in the genus *Fusarium* has been well described and includes 16 genes that encode biosynthetic enzymes and regulatory and transport proteins. Functions of genes in fumonisin biosynthesis have been determined in Fusarium verticillioides (26), and the number, order, and genomic orientation of the *Fum* genes are known in F. proliferatum and F. oxysporum (27–29). Sequences flanking the *Fum* gene cluster differ among species, however, indicating that the cluster’s genomic location is species dependent (26). Reports of fumonisin production on cracked corn (10, 14, 20, 30, 31) by some strains of F. temperatum and F. subglutinans are inconsistent with reported genetic capabilities for fumonisin biosynthesis by these species as sequenced strains of both F. subglutinans and F. temperatum lack one or more of the *Fum* genes required for fumonisin biosynthesis (26, 28, 32).

The objectives of this study were to further test the ability of these species to synthesize beauvericin and/or fumonisin with definitive chemical tests of strains not cultured on cracked corn and genetic analyses of additional strains. Our working hypotheses were the following: (i) that no strains of either species could synthesize fumonisin, (ii) that F. temperatum strains, but not those of F. subglutinans, could synthesize beauvericin, and (iii) that the chemical phenotypes would be consistent with the genomic sequence genotypes. The study advances the field by providing new insights into the toxigenic potential of these species and enabling more accurate estimation of the risks they pose to the food and feed products they might contaminate.

(Portions of this work are based on studies conducted by M. V. Fumero in partial fulfillment of the requirements for a Ph.D. from the National University of Rio Cuarto, Rio Cuarto, Cordoba, Argentina [March 2017].)

## RESULTS

### Strain isolation and identification.

**FIG 1:**
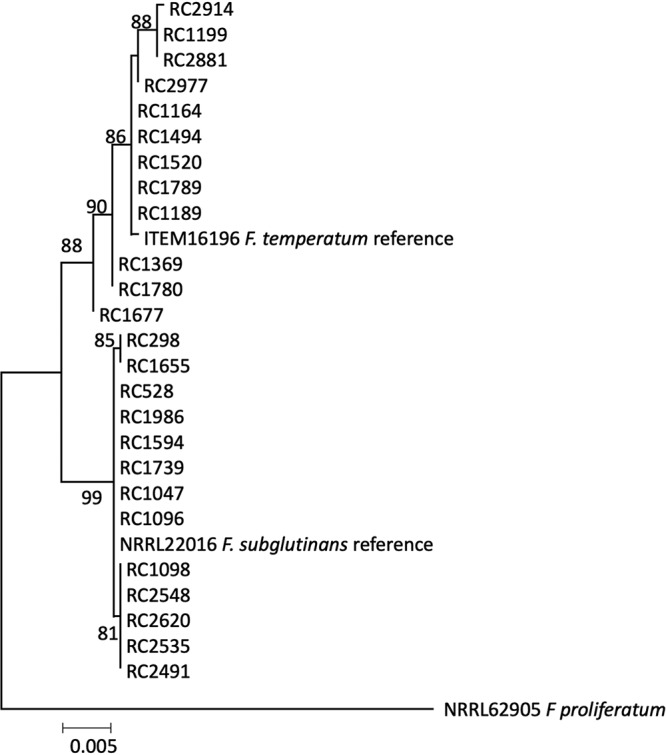
Phylogenetic tree derived from combined DNA sequences of *Tub2*, *Tef1*, and *Rpb2*. The evolutionary history was inferred using the maximum likelihood method. Numbers on branches indicate bootstrap values based on 1,000 pseudoreplicates. RC strains are from the strain collection at the National University of Rio Cuarto; ITEM strains are from ISPA, Bari, Italy; NRRL strains are from the USDA-ARS Culture Collection at the National Center for Agricultural Utilization Research, Peoria, IL.

Twenty-five *Fusarium* strains from Argentina (Table 1) were identified to species level in a maximum likelihood (ML) phylogenetic analysis of a three-gene combined data set, including sequences of reference strains from related species, with F. proliferatum NRRL 62905 as the outgroup (Fig. 1). Twelve strains were contained within a well-supported clade (bootstrap value, 88) that included the F. temperatum reference strain ITEM 16196 (MUCL 52463) (4). The remaining 13 strains were contained within a second well-defined clade (bootstrap value, 99) that included the F. subglutinans reference strain NRRL 22016 (Fig. 1).

**TABLE 1:** Strain identification, geographic origin, NCBI sequence number, and mycotoxin profile^*a*^

Strain	Geographic origin^*b*^	Beauvericin production^*c*^	NCBI accession no. for:		
			*Tef1*	*Tub2*	*Rpb2*
F. temperatum strains					
ITEM 16196^*d*^	Belgium	ND	MT345561	MT345559	MT345560
RC 1164	Tartagal	+	MT337672	MT337622	MT337647
RC 1189	Tartagal	+	MT337676	MT337626	MT337651
RC 1199	Tartagal	+	MT337669	MT337619	MT337644
RC 1369	NOA 1	+	MT337677	MT337627	MT337652
RC 1494	NOA1	−	MT337673	MT337623	MT337648
RC 1520	NOA1	−	MT337674	MT337624	MT337649
RC 1677	SEBA	+	MT337679	MT337629	MT337654
RC 1780	NOA1	−	MT337678	MT337628	MT337653
RC 1789	NOA1	+	MT337675	MT337625	MT337650
RC 2881	NOA1	+	MT337670	MT337620	MT337645
RC 2914	NOA1	+	MT337668	MT337618	MT337643
RC 2977	NOA1	+	MT337671	MT337621	MT337646
F. subglutinans strains					
NRRL 22016	USA	ND	HM057336	U34417	JX171599
RC 298	SEBA	−	MT337655	MT337605	MT337630
RC 528	Lajitas	−	MT337657	MT337607	MT337632
RC 1047	SEBA	−	MT337661	MT337611	MT337636
RC 1096	SEBA	−	MT337662	MT337612	MT337637
RC 1098	SEBA	−	MT337663	MT337613	MT337638
RC 1594	SEBA	−	MT337659	MT337609	MT337634
RC 1655	SEBA	−	MT337656	MT337606	MT337631
RC 1739	SEBA	−	MT337660	MT337610	MT337635
RC 1986	SEBA	−	MT337658	MT337608	MT337633
RC 2491	Lajitas	−	MT337667	MT337617	MT337642
RC 2535	Lajitas	−	MT337666	MT337616	MT337641
RC 2548	Lajitas	−	MT337664	MT337614	MT337639
RC 2620	Lajitas	−	MT337665	MT337615	MT337640

a No strain produced fumonisin when cultured on PDA. ND, no data from this study.

b The SEBA region contains three locations in southeast Buenos Aires province, with a 13.9°C (8.2 to 20.2°C) mean annual temperature and 550 to 900 mm of annual precipitation. Tartagal and Lajitas are locations in the Salta province, with a 21.1°C (14.3 to 26.4°C) mean annual temperature and 650 to 800 mm of annual precipitation and 20.4°C (16.7 to 28.1°C) mean annual temperature and 500 to 800 mm of annual precipitation, respectively. NOA1 contains four locations across Quebrada de Humahuaca in the Jujuy province, with an 11.7°C (5.1 to 16.3°C) mean annual temperature and 400 mm of annual precipitation.

c Range, 7 to 400 μg/kg; mean production, 71 μg/kg; median production, 11 μg/kg.

d Information on ITEM strains is available on line at http://www.ispa.cnr.it/Collection/.

### Genome analyses.

**(i) Genome assemblies.** We generated genome assemblies for two strains of F. subglutinans (RC 298 and RC 528) and one strain of F. temperatum (RC 2914) (Table 2). For F. temperatum RC 2914, ∼7.7 million reads were assembled in 720 scaffolds, for a total length of 42.5 Mb when only scaffolds of ≥10 kb in length were included. The scaffold *N*_50_, i.e., the length of the shortest scaffold such that 50% of the assembly is found in scaffolds of this length or longer, was 334 kb, and the longest scaffold was 1.5 Mb. The average coverage was 53×. Two scaffolds were retained for analysis of the *Bea* and *Fum* clusters.

**TABLE 2:** Genome statistics

Parameter	Value for the parameter in:		
	F. temperatum RC 2914	F. subglutinans RC 298	F. subglutinans RC 528
Scaffold *N*_50_ (bp)	334,266	228,189	203,510
Total no. of scaffolds	720	4,088	1,418
Longest scaffold (bp)	1,464,565	974,989	997,454
Total no. of bases in scaffolds of ≥1 kb in length	43,206,368	50,560,826	43,931,993
Total no. of bases in scaffolds of ≥10 kb in length	42,527,692	49,665,874	43,037,708
Genomic read fold coverage	53.3	100.7	60.9

For F. subglutinans RC 298, ∼17 million reads were assembled in 4,088 scaffolds, for a total length of 49.7 Mb when only scaffolds of ≥10 kb in length were included. The scaffold *N*_50_ was 228 kb, and the largest scaffold was 975 kb. The average coverage was 101×. Two scaffolds were retained for analysis of the *Bea* and *Fum* clusters. Finally, for F. subglutinans RC 528, ∼9 million reads were assembled in 1,418 scaffolds, for a total length of 43 Mb when only scaffolds of ≥10 kb in length were included. The scaffold *N*_50_ was 204 kb, and the largest scaffold was 997 kb. The average coverage was 61×. Again, two scaffolds were retained for analysis of the *Bea* and *Fum* clusters.

**FIG 2:**
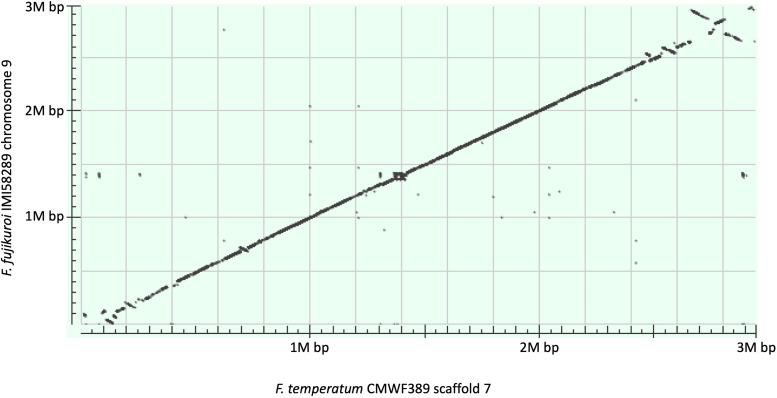
Comparison between chromosome 9 of Fusarium fujikuroi IMI 58289 (GenBank accession number NC_036630.1) and scaffold 7 of Fusarium temperatum CMWF 389 (LJGR01000007.1). Dot plot alignments show good synteny across both sequences but also some inverted regions and gaps.

**FIG 3:**
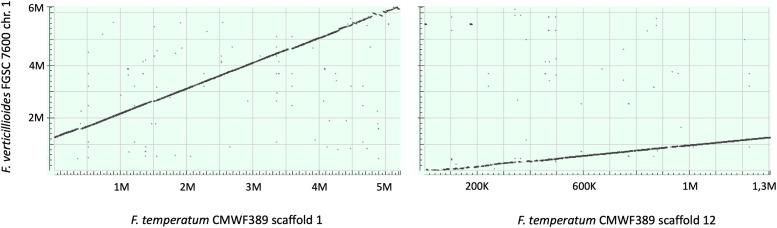
Comparison between chromosome 1 of Fusarium verticillioides FGSC 7600 (GenBank accession number NC_031675.1) and scaffolds 1 and 12 of Fusarium temperatum CMWF 389 (LJGR01000001.1 and LJGR01000012.1). Dot plot alignments show that both scaffolds 1 and 12 almost completely cover chromosome 1. Dot plot alignments show good synteny across sequences but also some inverted regions and gaps.

**(ii) Genomic context of contigs containing the beauvericin and fumonisin clusters.** Dot plot analysis between chromosome 9 of F. fujikuroi IMI 58289 (Ffuj_Chr9), where a complete *Bea* cluster is located, and scaffold 7 of F. temperatum CMWF 389 (Ftemp_Scaff7) identified sequences of almost the same length with complete synteny. Thus, Ftemp_Scaff7 probably is orthologous to chromosome 9 predicted for F. fujikuroi (Fig. 2). Dot plot analysis between chromosome 1 of F. verticillioides FGSC 7600 (Fv_Chr1), where the *Fum* cluster is located, and two scaffolds of F. temperatum CMWF 389, scaffold 1 (Ftemp_Scaff1) and scaffold 12 (Ftemp_Scaff12), had very good synteny. Thus, chromosome 1 of F. verticillioides is orthologous to Ftemp_Scaff1 and Ftemp_Scaff12 (Fig. 3).

**FIG 4:**
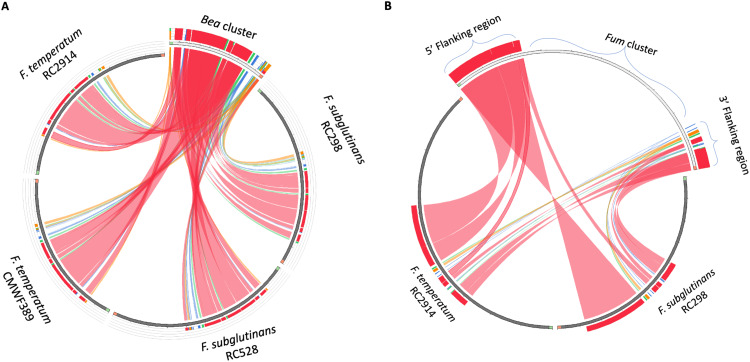
Circos plots showing the synteny across *Bea* (left) and *Fum* (right) clusters, a chromosome segment from the F. temperatum reference, and contigs from the new genome assemblies. Ribbons connecting the sequences represent local alignments produced by the BLAST algorithm. The ribbon colors indicate percentage identity as follows: blue, <50%; green, <75%; orange, <99%; and red, ≥99%. (A) Ideogram built using the Circoletto program comparing sequences of the *Bea* cluster of F. fujikuroi IMI 58289 (segment that protrudes at the upper right of the circle) with the newly sequenced genomes of F. subglutinans RC 298, RC 528, F. temperatum RC 2914, and the South African reference strain CMWF 389 (sections of the circle in dark gray). Each section represents sequence from an individual strain. (B) Ideogram built using the Circoletto program showing a comparison between the *Fum* cluster and related flanking regions (5ʹ flanking region, ZNF1 and ZBD1; 3ʹ flanking region, ORF20 and ORF21) of F. verticillioides FGSC 7600 (segment that protrudes at the upper right of the circle) and newly sequenced genomes of F. subglutinans RC 298 and F. temperatum RC 2914 (sections of the circle in dark gray). In order to show the absence of the *Fum* cluster and the adjacency between the flanking regions in greater detail, only one strain of each species is included in the graph. In both F. subglutinans and F. temperatum the *Fum* cluster 5ʹ and 3ʹ flanking regions are directly adjacent in their respective contigs, indicating the absence of the *Fum* cluster. Note the twists in the ribbons here, indicating inverted orientations of multiple segments of these flanking regions.

Circos plot analysis with the complete *Bea* cluster from Ffuj_Chr9 and portions of Ftemp_Scaff7 and the three newly sequenced *Bea*-containing contigs shows that the *Bea* cluster is complete in both F. temperatum and F. subglutinans (Fig. 4A).

Circos plot analysis with the complete *Fum* cluster from F. verticillioides chromosome 1 and portions of contigs from the three newly sequenced strains shows a gap in the synteny. Thus, both F. temperatum and F. subglutinans lack most of the genes normally found in this biosynthetic cluster (Fig. 4B).

### Beauvericin cluster.

**FIG 5:**
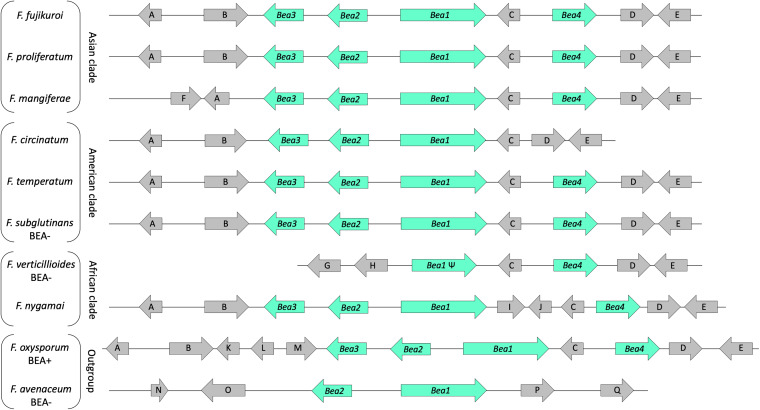
Organization of the *Bea* gene cluster and flanking genes. The arrows represent the indicated genes while the direction of the arrow shows direction of transcription. Blue arrows indicate known *Bea* cluster genes (21). Gray arrows indicate genes that flank the *Bea* cluster. Genes A, B, C, D, F, G, H, I, J, K, L, M, N, and O share >70% identity with FFUJ_09292, FFUJ_09293, FFUJ_09297, FFUJ_09299, FFUJ_09291, FFUJ_09286, FFUJ_09287, FNYG_14765, FNYG_14764, FOXG_11842, FOXG_11843, FOXG_11844, FFUJ_08099, and FFUJ_08100, respectively. Genes E, P, and Q share <50% identity with FFUJ_09300, FOZG_00061, and FPRN_10819, respectively. Ψ, pseudogene (nonfunctional). Strains used are Fusarium avenaceum Fa 05001, Fusarium circinatum FSP 34, Fusarium fujikuroi IMI 58289, Fusarium mangiferae MRC 7560, Fusarium nygamai MRC 8546, Fusarium oxysporum 4287, Fusarium proliferatum NRRL 62905, Fusarium subglutinans RC 298, Fusarium temperatum RC 2914, and Fusarium verticillioides FGSC 7600.

The entire *Bea* cluster (21) is present in the F. subglutinans and F. temperatum strains sequenced in the current study, as well as in several other closely related species that produce beauvericin, e.g., F. fujikuroi, F. mangiferae, Fusarium nygamai, F. oxysporum, and F. proliferatum (Fig. 5). In Fusarium circinatum FSP 34, the Zn(II)_2_Cys_6_ transcription factor (FFUJ_09298), encoded by *Bea4*, is absent, and this gene also is missing in the other two F. circinatum genomes in GenBank (strains GL 1327 and KS 17).

**FIG 6:**
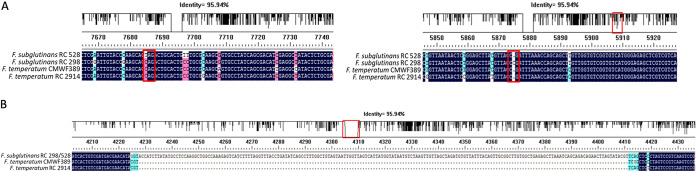
(A) Single-base mutations in RC 298 and RC 528 that could contribute to a nonfunctional *Bea1* (NRPP) gene. (B) Presence of the 184-bp insertion in both F. subglutinans genomes analyzed in this study. Red squares indicate genomic locations in the alignment where the indicated polymorphisms are observed.

Complete and functional BEA2, BEA3, and BEA4 proteins are predicted for all three genomes assembled in this study. The *Bea1* gene encoding the nonribosomal peptide synthase NRPS22 is predicted to produce a functional protein in both F. temperatum strains (RC 2914 and CMWF 389). In F. subglutinans, the predicted protein is apparently nonfunctional in strain RC 528 due to a single nucleotide polymorphism (SNP) resulting in a premature stop codon (CAG → TAG transition; the SNP is underlined). This transition occurs at nucleotide position 7685 (relative to the *Bea1* sequence from F. fujikuroi IMI 58289), where position 1 coincides with the start of the reading frame, i.e., the adenine of the ATG start codon. In strain RC 298, there is an insertion of a single cytosine at position 5875 that results in a frameshift and premature truncation of the protein (Fig. 6).

Both of the F. subglutinans strains had a 184-bp insertion between nucleotides 4223 and 4416 (Fig. 6). If this insertion was transcribed, it would add 61 amino acids to the length of the protein and cause a frameshift in the downstream reading frame that would lead to premature truncation of the protein. *In silico* prediction programs exclude the 184-bp insertion region from the open reading frame and instead introduce novel introns to prevent the premature truncation of the protein due to in-frame stop codons within the insertion. This predicted gene transcript would still result in a large protein, but it is uncertain whether the resulting protein would function properly.

### Fumonisin cluster.

The entire *Fum* cluster was missing from the F. subglutinans and F. temperatum genomes, which is consistent with the reported inability of many strains of these species to produce fumonisins. We searched for portions of all 16 *Fum* cluster genes (26) but found no recognizable homologous sequences.

**FIG 7:**
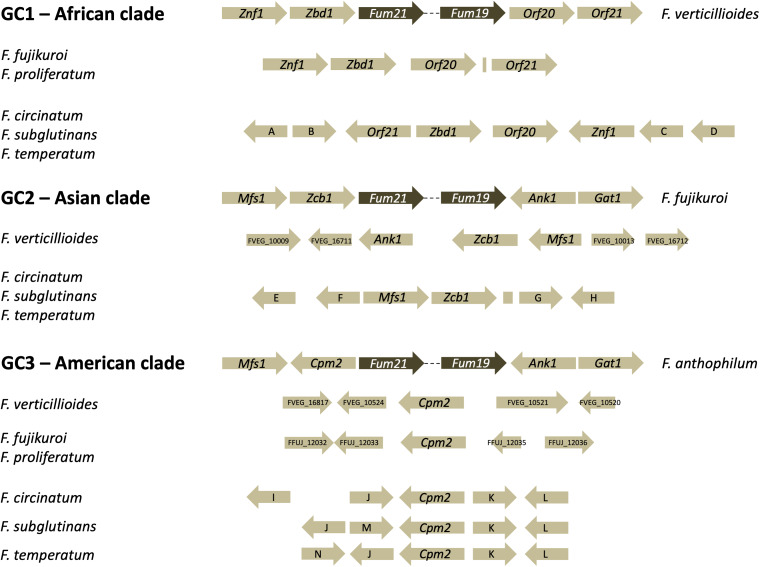
Organization of genes flanking the *Fum* cluster. The genes and the different genomic contexts (GC1, GC2, and GC3) were previously described by Proctor et al. (26). The *Fum* cluster is in different chromosomal locations in GC1, GC2, and GC3. Arrows represent the indicated genes while the direction of the arrow shows the direction of transcription. Only the marginal genes (*Fum19* and *Fum21*) of the *Fum* cluster are shown. Genes A, B, C, D, E, F, G, H, I, J, K, L, M, and N share >70% identity in blastp analysis with FVEG_00333, FVEG_00334, FVEG_00312, FVEG_00311, FFUJ_09236, FFUJ_09237, FFUJ_09258, FFUJ_09259, FVEG_10515, FFUJ_12036, FVEG_10524, FVEG_10525, FFUJ_12035, and FOXB_15017, respectively. The strains examined in this study are Fusarium circinatum FSP 34 (NCBI assembly accession number GCA_000497325), Fusarium fujikuroi IMI 58289 (GCA_900079805), Fusarium proliferatum NRRL 62905 (GCA_900029915), Fusarium subglutinans RC 298, Fusarium temperatum RC 2914, and Fusarium verticillioides FGSC 7600 (GCA_000149555).

Fusarium subglutinans and F. temperatum are members of the American clade of the F. fujikuroi species complex (FFSC). Some members of this clade, e.g., Fusarium anthophilum and Fusarium bulbicola, can produce fumonisins and carry the *Fum* biosynthetic gene cluster (26, 32, 33). We queried our newly generated genomes and those of some other members of the FFSC with genes that flank the *Fum* cluster in species from all three clades of the FFSC (26). In all cases, the *Fum* cluster was absent from F. subglutinans and F. temperatum. Instead, we found one of four flanking genes (*Cpm2*) from the American clade species and two of four genes from Asian clade species (*Mfs1* and *Zcb1*). We also found all four genes queried from African clade species (*Znf1*, *Zbd1*, *Orf20*, and *Orf21*) although the orientations and order of *Orf21* and *Znf1* were different in F. subglutinans and F. temperatum from those in F. verticillioides (Fig. 7).

### Mycotoxin production.

Nine of the 12 strains identified as F. temperatum produced beauvericin at levels ranging from 7 to 400 μg/kg (mean, 71 μg/kg; median, 11.3 μg/kg), whereas no F. subglutinans strains produced beauvericin. None of the 25 strains examined produced fumonisin B_1_ (FB_1_) on potato dextrose agar (PDA) (Table 1).

## DISCUSSION

Fusarium subglutinans and F. temperatum are well known as preharvest fungal pathogens that cause maize stalk and ear rot and are closely related species that can be easily misidentified (4). Strains of these species can produce a variety of mycotoxins (2, 8, 10, 14, 30, 31, 34). However, reports of mycotoxin production by these species are not consistent (14, 20), leading to confusion regarding the specific mycotoxin profile that they possess. This confusion can result in underestimation or overestimation of the mycotoxin-associated risk posed by foods and feeds contaminated with these fungi. It also makes it very difficult to develop effective pre- and postharvest strategies for monitoring and managing mycotoxin contamination.

There are multiple reports of fumonisin production (10, 14, 30, 31) and nonproduction (2, 14, 34, 35) by F. subglutinans groups 1 and 2, which are now F. temperatum and F. subglutinans, respectively. The lack of all or parts of the *Fum* gene cluster in some strains of both species has been reported on multiple occasions (26, 27, 32). In our study, we found that some genomes of both species lacked the entire *Fum* cluster and that the insertion sites across species in the FFSC that contain part or all of the *Fum* gene cluster are not well conserved. For example, in Fusarium musae, a sister species of F. verticillioides that cannot produce fumonisins (36, 37), only remnants of the *Fum21* and *Fum19* genes, at the opposite ends of the cluster, remain along with some of the flanking genes. The deletions and rearrangements we detected in genomic regions where the *Fum* cluster is inserted in other species suggest that changes related to *Fum* cluster insertion/deletion are not simple events and could have occurred in more than one step at more than one time.

In contrast with fumonisins, there is a general consensus that strains of F. subglutinans do not produce beauvericin but that some strains of F. temperatum do (10–12, 14–16, 18). In the present study, we found that 75% of the F. temperatum strains analyzed could produce beauvericin but that none of the strains of F. subglutinans could. Unlike the *Fum* cluster, however, the molecular basis for the differences between toxin-producing and toxin-nonproducing strains was not previously known.

The *Bea* gene cluster contains four genes, *Bea1* to *Bea4*, of which two, *Bea1* and *Bea2*, are essential for beauvericin production, while the other two, *Bea3* and *Bea4*, encode proteins that repress beauvericin production (21). BEA4 is not essential for beauvericin production since F. circinatum can synthesize beauvericin (5, 38–40) but lacks the gene encoding this protein (21). In fact, deletion of *Bea4* could potentially increase beauvericin production by removing a layer of repressive regulation.

The *Bea1*-encoded nonribosomal polypeptide (NRPP) synthetase required for biosynthesis of the cyclic depsipeptide beauvericin was first described in the fungus Beauveria bassiana over 50 years ago (41, 42) and later confirmed in F. circinatum, F. oxysporum, F. proliferatum, and F. fujikuroi (21, 23, 39, 43, 44). Molecular organization of the *Bea* gene cluster has not been analyzed as extensively as has the *Fum* gene cluster. The genomic organization of the *Bea* clusters in F. subglutinans, F. temperatum, F. circinatum, F. proliferatum, F. fujikuroi, F. mangiferae, and F. nygamai is consistent with respect to gene order, direction of transcription, and genomic context; however, there are differences in individual gene coding sequences.

The available F. temperatum genomes are all from beauvericin-producing strains and harbor intact, functional sequences for all of the *Bea* genes in the cluster. All F. subglutinans genomes carry functional *Bea2* to *Bea4* genes. The *Bea1* gene appears to encode a nonfunctional protein in both of the analyzed sequences from F. subglutinans. Both of these genomes contain a 184-bp insertion at position 4233. This insertion results in a protein projected to be nonfunctional, whether it alters splicing and intron arrangement or is read as a coding part of the gene. Each strain carries a second, but different, mutation that also inactivates the protein. In RC 298, there is a single nucleotide insertion at position 5686 that introduces a frameshift resulting in a stop codon 120 bp further downstream (position 5806) that should prevent translation of a full-length protein. In RC 528, a single nucleotide substitution at position 7685 results in a premature stop codon 1,907 bp upstream of the 3ʹ end of the coding region.

The accumulation of only a couple of loss-of-function mutations in *Bea1* suggests that the process of its inactivation began relatively recently. As both strains have the 184-bp insertion, this genomic change probably occurred first. Assuming that this insertion prevents beauvericin accumulation, then subsequent mutations in genes required exclusively for beauvericin biosynthesis would occur without selection acting against them. Thus, the longer a gene has been nonfunctional, the more mutations it should have accumulated in its coding sequence. After the insertion occurred, flawed transcripts might still produce altered proteins. If so, secondary mutations, such as the *Bea1* single nucleotide insertion or substitution we observed, could have been selected for to reduce the production of proteins with toxic effects or to reduce the energetic costs due to transcription and translation of nonfunctional genes, speeding the rate at which mutations accumulate (45–47). Given the difference in secondary mutations seen in the strains sequenced, other strains that do not produce beauvericins could well have other mutations in *Bea1* or elsewhere that prevent beauvericin biosynthesis. Yet the few loss-of-function mutants found in either of the two sequenced strains support a recent *Bea1* inactivation.

Analysis of transcripts from the mutated gene could provide insights into how F. subglutinans has managed the 184-bp insertion in this gene. For example, are the novel introns predicted in the *in silico* analysis present? Or is the entire insertion translated, which would result in a single-base frameshift mutation? The F. temperatum strains that do not produce beauvericin could be of interest as well. Do they carry the 184-bp insertion and either of the other mutations observed in the F. subglutinans genomes? Or is their inability to produce beauvericin due to mutations elsewhere in the *Bea* cluster or the strains’ genomes?

The nature of the genomic changes that disrupt mycotoxin production plays a role in the potential development of diagnostic PCR tests for whether strains could potentially produce fumonisins or beauvericin. Strains of both species would be negative if any primer pairs designed to amplify any portion of the *Fum* cluster were used as the entire cluster is missing from the available genomes. A similar test for the potential to produce beauvericin is more problematic. Both species have all of the genes in the *Bea* cluster, and the genes *Bea2* to *Bea4* are predicted to be intact and functional. Thus, any successful DNA-based assay would need to be specific to *Bea1*. To detect the aberrant F. subglutinans versions of these genes, the assay could have primers that result in a larger fragment due to the 184-bp insertion or have one primer based on a unique sequence within the inserted region. Tests that detected a secondary SNP or the presence of the insertion also could identify nonfunctional alleles. Other PCR tests involving *Bea1*, i.e., simply detecting the presence of the gene or a portion of it, would be unable to distinguish a functional version of the gene from the nonfunctional version seen in F. subglutinans. Depending on the reason for the inability of the three F. temperatum strains to produce beauvericin, this assay could become even more complex.

In conclusion, we found that 25 strains of F. subglutinans and F. temperatum from Argentina could not synthesize fumonisins. The genomic basis for the lack of fumonisin production is presumably the complete absence of the genes in the *Fum* cluster, given the available genome sequences. As some F. temperatum strains are reported to produce fumonisins (14, 20), however, sequences of genomes from these strains are needed to understand the complexities of mycotoxin production in this species. We also confirmed that all tested strains of F. subglutinans and a subset of F. temperatum strains cannot synthesize beauvericin and note that the lack of beauvericin production cannot be used to definitively identify a strain as F. subglutinans. The *Bea* cluster was organized consistently in terms of location, gene order, and direction of transcription in F. circinatum, F. fujikuroi, F. subglutinans, and F. temperatum. Potential similarities in the *Bea1* sequences from strains of F. subglutinans and the non-toxin-producing strains of F. temperatum could show whether the initial inactivation event preceded the separation of F. subglutinans and F. temperatum as separate species. Since the NRPP responsible for enniatin synthesis differs in only a few amino acids from the NRPP responsible for beauvericin synthesis (48), it will be interesting to determine if events that prevent enniatin synthesis are similar to those that prevent beauvericin synthesis. Our study provides a firm genetic and physiological base on which future studies of these toxins can be built.

## MATERIALS AND METHODS

### Fungal isolates.

Strains of *Fusarium* were recovered from maize harvested in four regions of Argentina where the presence of F. subglutinans and F. temperatum had previously been reported (14, 49). Maize grains were incubated on pentachloronitrobenzene (PCNB) medium (24), and the resulting *Fusarium* colonies were purified by subculturing single microconidia from them. Morphological identifications were made following growth on homemade (24) and commercial (Biolife, Milan, Italy) potato dextrose agar (PDA), carnation leaf agar (CLA) (24), and Spezieller Nährstoffarmer agar (SNA) (24) for 10 days at 25°C under 12-h alternating periods of light (combination of cool white and black lights) and darkness. Colony morphology was evaluated on PDA. Spore morphology was evaluated using spores from colonies growing on CLA or SNA. Strains with the morphological characteristics of F. subglutinans described by Leslie and Summerell (24) were selected for DNA-based identification and further study.

### DNA-based identification of fungal isolates.

**(i) DNA extraction.** Twenty-five strains with morphology consistent with that of F. subglutinans were selected for DNA-based identification. Isolates were grown on PDA for 2 days at 25°C in the dark. Fresh mycelia were collected by scraping the plate surface and collecting the mycelia in 2-ml tubes. Total genomic DNA was extracted from 30 mg of freeze-dried and ground mycelia by using a Wizard Magnetic DNA Purification System for Food kit (Promega, Madison, WI) according to the manufacturer’s protocol. DNA was quantified in a NanoDrop spectrophotometer, and the DNA concentration was adjusted to 20 ng/μl for PCR amplifications.

**(ii) Gene sequencing.** Portions of three housekeeping genes, encoding β-tubulin (*Tub2*), translation elongation factor (*Tef1*), and the second largest subunit of RNA polymerase II (*Rpb2*), were used for species identification. Previously described PCR conditions and primers were used for each gene: BT2a/BT2b for *Tub2* (50), EF1/EF2 for *Tef1* (51), and 5F/7cR for *Rpb2* (52). PCR amplicons were cleaned before sequencing with EXO/FastAp (exonuclease I, Escherichia coli/FastAP thermosensitive alkaline phosphatase; ThermoFisher Scientific Baltics, Vilnius, Lithuania) to hydrolyze excess primers and nucleotides. Both strands were sequenced with a BigDye Terminator, version 3.1, cycle sequencing ready reaction kit. Sequence reaction products were purified by gel filtration through Sephadex G-50 (5%) (Amersham Pharmacia Biotech, Piscataway, NJ) and analyzed on a 3730xl DNA analyzer (Applied Biosystems, Foster City, CA). The software package Bionumerics, version 5.1 (Applied Maths, Sint-Martens-Latem, Belgium), was used to align the two DNA strands and edit the sequence. Edited sequences were compared with sequences in the *Fusarium*-ID (53) and GenBank databases. The phylogenetic species identity of each field strain was assigned to the species of database strains when sequence identity was >98%. NCBI accession numbers for *Tef1*, *Tub2*, and *Rpb2* sequences for each strain are listed in Table 1.

**(iii) Phylogenetic analyses.** DNA sequences consisting of partial sequences of *Tub2*, *Tef1*, and *Rpb2* were concatenated and then aligned with ClustalW. The resulting combined data set was analyzed with the maximum likelihood algorithm implemented in IQ-TREE (54) with the Tamura-Nei substitution model (55) and 1,000 bootstrap replicates (56). The alignment was deposited in TreeBASE (https://www.treebase.org/treebase-web/search/studySearch.html) under study number 25708.

### Gene cluster analysis.

**(i) DNA extraction for whole-genome sequencing.** The genomes of two F. subglutinans strains (RC 298 and RC 528) and one F. temperatum strain (RC 2914) were sequenced. Each strain was cultivated in 50 ml of complete medium and incubated on an orbital shaker at 150 rpm for 2 days at 25°C (24). Mycelia were collected following vacuum filtration through nongauze milk filter disks (KenAG, Ashland, OH) and stored at –20°C in 2-ml tubes. Frozen mycelia were lyophilized (Labconco Corporation, Kansas City, MO), added to microcentrifuge tubes containing two 4.5-mm zinc-plated steel beads (Daisy BBs, Rogers, AR), and ground to a fine powder in a mixer mill (Verder Scientific, Retsch, Germany). Genomic DNA was isolated by following a modified cetyltrimethylammonium bromide (CTAB) protocol (24). The resulting DNA was resuspended in Tris-EDTA (TE) buffer (pH 8.0) and stored at –20°C. DNA quality was checked by separation in a 1% agarose gel. DNA concentration was measured with a Quant-iT PicoGreen double-stranded DNA (dsDNA) assay kit (Life Technologies, Carlsbad, CA), and the results were read in a Synergy H1 hybrid reader (BioTek Instruments, Inc., Winooski, VT). The DNA was diluted to a final concentration of 100 ng/μl.

**(ii) Genome sequencing and assembly.** Three paired-end libraries (one for each selected strain) were constructed and sequenced with an Illumina MiSeq sequencer using paired-end 300-bp reads at the Kansas State University Integrated Genomics Facility. Genomes were assembled into contigs by using the de Bruijn graph-based algorithm implemented in the DISCOVAR *de novo* software from the Broad Institute, Cambridge, MA (https://software.broadinstitute.org/software/discovar/blog/) with the default parameters (*k*-mer of 200). Fastq files were converted to BAM files with the tools in Picard, version 2.12.1 (http://broadinstitute.github.io/picard). Though the DISCOVAR *de novo* assembly does not contain long-range scaffolding information, the sequences represented by these fastq files are technically scaffolds due to the presence of some stretches of Ns that bridge small gaps in read coverage. We refer to them as scaffolds although they are functionally more similar to contigs from other assemblies.

**(iii) Screening for the presence of beauvericin and fumonisin biosynthetic gene clusters in the newly sequenced genomes of Fusarium subglutinans and Fusarium temperatum.** The newly sequenced genomes F. subglutinans (RC 298 and RC 528) and F. temperatum (RC 2914), as well as the publicly available F. temperatum genome CMWF 389 (57), were evaluated for the presence of genes involved in beauvericin and fumonisin production. Genes from the *Bea* cluster in F. fujikuroi (FFUJ_09294 to FFUJ_09298) (21) were used as probes in a blastn analysis of individual genome sequence databases in CLC Genomics Workbench, version 8.0 (CLC Bio-Qiagen, Aarhus, Denmark). Sequences of *Bea* genes from beauvericin-producing strains of F. circinatum FSP 34 (58), F. fujikuroi IMI 58289 (21, 59), F. mangiferae MRC 7560 (21), F. nygamai MRC 8546 (60), F. oxysporum 4287 (43), F. proliferatum NRRL 62905 (21), and beauvericin-nonproducing strains of F. verticillioides FGSC 7600 (61) and F. avenaceum Fa 05001 (48) were identified in GenBank and included in the comparative analysis.

The same blastN analysis protocol was used for the *Fum* gene cluster but with the predicted F. verticillioides *Fum* gene cluster serving as the reference (FVEG_00316 to FVEG_00329) (27, 62). For the *Fum* cluster, the analysis was extended to regions flanking the cluster by including the genes described by Proctor et al. (26).

Annotation of the *Bea* biosynthetic genes and *Fum* flanking genes present in the newly sequenced genomes of F. subglutinans and F. temperatum was done manually, with the gene prediction tools Augustus (63) and FGENESH (64). The locations of coding sequences and introns were determined by comparison with the publicly available annotated sequences of the reference strains.

**(iv) Genomic context of newly sequenced contigs containing clusters of interest.** The genomic contexts of the putative *Bea* and *Fum* clusters in the newly sequenced genomes of F. temperatum RC 2914 and F. subglutinans RC 298 and RC 528 were established. The F. temperatum CMWF 389 (57) genome assembly used as a reference is in the scaffold stage, so dot plots were used to compare these scaffolds with the well-annotated chromosomes of F. verticillioides FGSC 7600 (61) and F. fujikuroi IMI 58289 (59). The online tool Circoletto (http://tools.bat.infspire.org/circoletto/) was run with default parameters (65). The resulting circular plots provide a global view of the sequence similarity between the *Bea* and *Fum* gene clusters and flanking regions from reference genomes and the newly sequenced contigs of F. subglutinans and F. temperatum. This software also was used to verify that contigs with blastn hits contained complete sequences of the clusters of interest, or the flanking regions, and to display aspects of the alignments, such as sequence rearrangements and percent identity.

### Mycotoxin analysis.

**(i) Beauvericin and fumonisin B_1_ (FB_1_) production *in vitro*.** Mycotoxins were produced on PDA, as previously described for *Fusarium* (66). Plates were centrally inoculated with 3-mm-diameter mycelial plugs from the edges of 7-day-old SNA cultures. Inoculated plates were incubated for 15 days in darkness at 25°C. Each plate was inoculated in duplicate. This experiment was performed once.

**(ii) Chemicals and preparation of standards.** All solvents (high-performance liquid chromatography [HPLC] grade) were purchased from VWR International SRL (Milan, Italy). Ultrapure water was produced by a Millipore Milli-Q system (Millipore, Bedford, MA). Beauvericin standards (purity of >99%) were purchased from Sigma-Aldrich (Milan, Italy), and FB_1_ was from Biopure (Romer Labs Diagnostic GmbH, Getzersdorf, Austria). Standard stock solutions (1 mg/ml) were prepared by dissolving the solid commercial toxin standards in methanol. For working solutions of beauvericin, some of the methanol stock solution was dried under a nitrogen stream at 50°C and reconstituted with methanol-water (70:30, vol/vol). Standard solutions for ultraperformance liquid chromatography (UPLC) calibration were prepared by using different concentrations in a range of 0.02 to 40.00 μg/ml. Working stock solutions of FB_1_ were prepared by drying some of the stock solution under a nitrogen stream and reconstituting it with acetonitrile-water (1:1, vol/vol). Standard solutions for UPLC calibration were prepared by using different concentrations in a range of 0.01 to 1.00 μg/ml. Standard solutions were stored at –20°C and warmed to room temperature (∼20 to 22°C) prior to use.

**(iii) Determination and confirmation of beauvericin production.** Ten grams of culture material was extracted with 15 ml of methanol on an orbital shaker (150 rpm) for 30 min. Six milliliters of the extract was evaporated to dryness under a stream of nitrogen at 40°C. The residue was dissolved in 1.5 ml of methanol-water (70:30, vol/vol) and filtered through a 0.2-μm-pore-size regenerated cellulose (RC) filter (Grace Davison Discovery Science, Columbia, MD). Ten microliters of the extract was injected into the full-loop injection system of an Acquity UPLC system (Waters, Milford, MA), equipped with an electrospray ionization (ESI) interface with a binary solvent manager, a sample manager, a column heater, a photodiode array, and quadrupole dalton (QDa) detectors. The analytical column was an Acquity UPLC BEH C_18_ (2.10 by 100 mm; 1.7-μm particle size) preceded by an Acquity UPLC in-line filter (0.20-μm pore size). The temperature of the column was set at 50°C. The flow rate of the mobile phase was set at 0.35 ml/min. The toxins were determined in both detectors, with the photodiode array set at 205 nm, and QDa mass detector (UPLC-PDA-QDa), without splitting. The mobile phase consisted of a gradient with two components: solvent A consisted of water with 0.1% formic acid, and solvent B consisted of acetonitrile with 0.1% formic acid. The initial composition 50:50 (A/B) was kept constant for 2 min; solvent B was then increased linearly to 75% in 8 min, followed by another linear increase to 80% in 2 min, and the composition was kept constant for 4 min. For column reequilibration, solvent B was linearly decreased to 50% in 1 min and then kept constant for 4 min. The limit of quantification (LOQ) of the method was 0.01 μg/kg.

For liquid chromatography mass spectrometry (LC/MS) analyses, the ESI interface was used in positive-ion mode, with the following settings: desolvation temperature of 600°C, capillary voltage at 0.80 kV, and sampling rate of 5 Hz. The mass spectrometer was operated in full-scan (600 to 800 *m/z*) and in single-ion recording (SIR) modes by monitoring the mass of beauvericin (784 *m/z*; elemental formula [M+H]^+^:C_45_H_57_N_3_O_9_). MassLynx, version 4.1, mass spectrometry software was used for data acquisition and processing. The retention time for beauvericin was ∼9.80 min. Beauvericin was quantified by measuring peak areas and comparing these values with a calibration curve obtained from standard solutions (48, 67, 68).

**(iv) Determination and confirmation of fumonisin production.** Ten grams of culture material was extracted with 15 ml of methanol-water (70:30, vol/vol) on an orbital shaker (150 rpm) for 60 min. Six milliliters of the extract was evaporated to dryness under a stream of nitrogen at 40°C. The residue was dissolved in 1.5 ml of acetonitrile-water (30:70, vol/vol), filtered with RC 0.2-μm-pore-size filters (Phenomenex, Torrance, CA), derivatized, as described below, and quantified by HPLC and fluorescence detection (FLD). To derivatize a sample, 50 μl of a sample extract was mixed with 50 μl of *o*-phthaladehyde (OPA) by shaking for 50 s in the HPLC autosampler of an Agilent 1100 equipped with a binary pump and a column thermostat set at 30°C. The 100-μl volume was injected by full-loop injection 3 min after addition of the OPA reagent for fumonisin analysis. The analytical column was a Symmetry Shield RP18 (4.6 by 150 mm, 5-μm particle size; Waters) with a guard column inlet filter (0.5-μm by 3-mm diameter; Postnova Analytics, Inc., Salt Lake City, UT). The mobile phase consisted of a binary gradient whose initial composition was 57% A (water-acetic acid, 99:1, vol/vol) and 43% B (acetonitrile-acetic acid, 99:1, vol/vol) and kept constant for 5 min. Solvent B was then linearly increased to 54% at 21 min, linearly increased again to 58% at 25 min, and finally kept constant for 5 min. The flow rate of the mobile phase was 0.80 ml/min. The fluorometric detector was set at an excitation wavelength of 335 nm and emission wavelength of 440 nm. Retention time for FB_1_ was 17 min. The LOQ of the method was 0.01 μg/kg.

Fumonisin B_1_ was confirmed by UPLC with an Acquity QDa mass detector. The chromatographic separation was performed on an Acquity UPLC BEH C_18_ column (2.1 by 100 mm; 1.7-μm particle size) preceded by an Acquity UPLC in-line filter (0.2-μm pore size). The temperature of the column was set at 50°C. The flow rate of the mobile phase was set at 0.4 ml/min. Solvent A was water, and solvent B was methanol, with both solvents containing 0.1% acetic acid. A gradient elution was used beginning with 90% A and 10% B. The gradient was changed from 10% to 50% solvent B in 10 min and kept constant for 4 min; it was linearly increased to 90% solvent B in 3 min, and then kept constant for 4 min. For column reequilibration, solvent B was decreased to 10% in 1 min and kept constant for 3 min.

For LC/MS analyses, the ESI interface was used in positive-ion mode, with the following settings: desolvation temperature of 600°C, capillary voltage of 0.80 kV, and sampling rate of 5 Hz. The mass spectrometer was operated in full-scan (100 to 800 *m/z*) and in single-ion recording (SIR) modes by monitoring the individual mass (FB_1_ 722.40 *m/z*). Retention time for FB_1_ was 16 min. Empower 2 software (Waters) was used for data acquisition and processing. The LOQ was 0.01 μg/ml for FB_1_ (48, 67, 68).

### Data availability.

Genome sequences were deposited in GenBank under accession numbers JAAIFR000000000 for RC 298, JAAIFQ000000000 for RC 528, and JAAIFN000000000 for RC 2914.

## References

[1] Moretti A, Logrieco A, Bottalico A, Ritieni A, Randazzo G, Corda P 1995 Beauvericin production by *Fusarium subglutinans* from different geographical areas. Mycol Res 99:282–286. doi:10.1016/S0953-7562(09)80899-X.

[2] Desjardins AE 2006 *Fusarium* mycotoxins: chemistry, genetics, and biology. APS Press, St. Paul, MN.

[3] Steenkamp ET, Wingfield BD, Desjardins AE, Marasas WF, Wingfield MJ 2002 Cryptic speciation in *Fusarium subglutinans*. Mycologia 94:1032–1043. doi:10.2307/3761868. 21156574

[4] Scauflaire J, Gourgue M, Munaut F 2011 *Fusarium temperatum* sp. nov. from maize, an emergent species closely related to *Fusarium subglutinans*. Mycologia 103:586–597. doi:10.3852/10-135. 21186324

[5] Jestoi M 2008 Emerging *Fusarium* mycotoxins: fusaproliferin, beauvericin, enniatins, and moniliformin: a review. Crit Rev Food Sci Nutr 48:21–49. doi:10.1080/10408390601062021. 18274964

[6] EFSA Panel on Contaminants in the Food Chain (CONTAM). 2014 Scientific opinion on the risks to human and animal health related to the presence of beauvericin and enniatins in food and feed. EFSA J 12:3802. doi:10.2903/j.efsa.2014.3802.

[7] Taevernier L, Wynendaele E, de Vreese L, Burvenich C, De Spiegeleer B 2016 The mycotoxin definition reconsidered towards fungal cyclic depsipeptides. J Environ Sci Health C Environ Carcinog Ecotoxicol Rev 34:114–135. doi:10.1080/10590501.2016.1164561. 26963720

[8] Moretti A, Mulè G, Ritieni A, Láday M, Stubnya V, Hornok L, Logrieco A 2008 Cryptic subspecies and beauvericin production by *Fusarium subglutinans* from Europe. Int J Food Microbiol 127:312–315. doi:10.1016/j.ijfoodmicro.2008.08.003. 18804303

[9] Munkvold GP, Logrieco A, Moretti A, Ferracane R, Ritieni A 2009 Dominance of group 2 and fusaproliferin production by *Fusarium subglutinans* from Iowa maize. Food Addit Contam Part A Chem Anal Control Expo Risk Assess 26:388–394. doi:10.1080/02652030802471239. 19680913

[10] Scauflaire J, Gourgue M, Callebaut A, Munaut F 2012 *Fusarium temperatum*, a mycotoxin-producing pathogen of maize. Eur J Plant Pathol 133:911–922. doi:10.1007/s10658-012-9958-8.

[11] Susca A, Villani A, Mulè G, Stea G, Logrieco AF, Moretti A 2013 Geographic distribution and multi-locus analysis of *Fusarium subglutinans* and *Fusarium temperatum* from maize worldwide, abstr P77, p 170 Abstr 12th Eur *Fusarium* Semin, Bordeaux, France, 11 to 16 May 2013.

[12] Boutigny AL, Scauflaire J, Ballois N, Ioos R 2017 *Fusarium temperatum* isolated from maize in France. Eur J Plant Pathol 148:997–1001. doi:10.1007/s10658-016-1137-x.

[13] Czembor E, Stępień Ł, Waśkiewicz A 2014 *Fusarium temperatum* as a new species causing ear rot on maize in Poland. Plant Dis 98:1001. doi:10.1094/PDIS-11-13-1184-PDN. 30708873

[14] Fumero MV, Reynoso MM, Chulze SN 2015 *Fusarium temperatum* and *Fusarium subglutinans* isolated from maize in Argentina. Int J Food Microbiol 199:86–92. doi:10.1016/j.ijfoodmicro.2015.01.011. 25647244

[15] Lanza FE, Mayfield DA, Munkvold GP 2016 First report of *Fusarium temperatum* causing maize seedling blight and seed rot in North America. Plant Dis 100:1019–1019. doi:10.1094/PDIS-11-15-1301-PDN.

[16] Robles-Barrios KF, Medina-Canales MG, Rodríguez-Tovar AV, Pérez NO 2015 Morphological and molecular characterization, enzyme production and pathogenesis of *Fusarium temperatum* on corn in Mexico. Can J Plant Pathol 37:495–505. doi:10.1080/07060661.2015.1113445.

[17] Varela CP, Casal OA, Padin MC, Martinez VF, Oses MS, Scauflaire J, Munaut F, Bande Castro MJ, Vázquez JM 2013 First report of *Fusarium temperatum* causing seedling blight and stalk rot on maize in Spain. Plant Dis 97:1252. doi:10.1094/PDIS-02-13-0167-PDN. 30722434

[18] Zhang H, Luo W, Pan Y, Xu J, Xu JS, Chen WQ, Feng J 2014 First report of *Fusarium temperatum* causing *Fusarium* ear rot on maize in Northern China. Plant Dis 98:1273–1273. doi:10.1094/PDIS-02-14-0124-PDN. 30699668

[19] Stepien L, Waskiewicz A 2013 Sequence divergence of the enniatin synthase gene in relation to production of beauvericin and enniatins in *Fusarium* species. Toxins 5:537–555. doi:10.3390/toxins5030537. 23486233 PMC3705277

[20] Fumero MV, Sulyok M, Chulze S 2016 Ecophysiology of *Fusarium temperatum* from maize in Argentina. Food Addit Contam Part A Chem Anal Control Expo Risk Assess 33:147–156. doi:10.1080/19440049.2015.1107917. 26535974

[21] Niehaus EM, Studt L, von Bargen KW, Kummer W, Humpf HU, Reuter G, Tudzynski B 2016 Sound of silence: the beauvericin cluster in *Fusarium fujikuroi* is controlled by cluster-specific and global regulators mediated by H3K27 modification. Environ Microbiol 18:4282–4302. doi:10.1111/1462-2920.13576. 27750383

[22] Munkvold GP 2017 *Fusarium* species and their associated mycotoxins, p 51–106. *In* Moretti A, Susca A (ed), Mycotoxigenic fungi. Humana Press, New York, NY. 10.1007/978-1-4939-6707-0_4 27924531

[23] Wu Q, Patocka J, Nepovimova E, Kuca K 2018 A review on the synthesis and bioactivity aspects of beauvericin, a *Fusarium* mycotoxin. Front Pharmacol 9:1338. doi:10.3389/fphar.2018.01338. 30515098 PMC6256083

[24] Leslie JF, Summerell BA 2006 The Fusarium laboratory manual. Blackwell Professional, Ames, IA.

[25] Paciolla C, Dipierro N, Mulè G, Logrieco A, Dipierro S 2004 The mycotoxins beauvericin and T-2 induce cell death and alteration to the ascorbate metabolism in tomato protoplasts. Physiol Mol Plant Pathol 65:49–56. doi:10.1016/j.pmpp.2004.07.006.

[26] Proctor RH, van Hove F, Susca A, Stea G, Busman M, van der Lee T, Waalwijk C, Moretti A, Ward TJ 2013 Birth, death and horizontal transfer of the fumonisin biosynthetic gene cluster during the evolutionary diversification of *Fusarium*. Mol Microbiol 90:290–306. doi:10.1111/mmi.12362. 23937442

[27] Proctor RH, Brown DW, Plattner RD, Desjardins AE 2003 Co-expression of 15 contiguous genes delineates a fumonisin biosynthetic gene cluster in *Gibberella moniliformis*. Fung Genet Biol 38:237–249. doi:10.1016/S1087-1845(02)00525-X. 12620260

[28] Proctor RH, Busman M, Seo JA, Lee Y-W, Plattner RD 2008 A fumonisin biosynthetic gene cluster in *Fusarium oxysporum* strain O-1890 and the genetic basis for B versus C fumonisin production. Fungal Genet Biol 45:1016–1026. doi:10.1016/j.fgb.2008.02.004. 18375156

[29] Waalwijk C, van der Lee T, de Vries I, Hesselink T, Arts J, Kema G 2004 Synteny in toxigenic *Fusarium* species: the fumonisin gene cluster and the mating type region as examples. Eur J Plant Pathol 110:533–544. doi:10.1023/B:EJPP.0000032393.72921.5b.

[30] Stępień Ł, Koczyk G, Waśkiewicz A 2013 Diversity of *Fusarium* species and mycotoxins contaminating pineapple. J Appl Genet 54:367–380. doi:10.1007/s13353-013-0146-0. 23572446 PMC3720990

[31] Wang JH, Zhang JB, Li HP, Gong AD, Xue S, Agboola RS, Liao YC 2014 Molecular identification, mycotoxin production and comparative pathogenicity of *Fusarium temperatum* isolated from maize in China. J Phytopathol 162:147–157. doi:10.1111/jph.12164.

[32] Proctor RH, Plattner RD, Brown DW, Seo JA, Lee Y-W 2004 Discontinuous distribution of fumonisin biosynthetic genes in the *Gibberella fujikuroi* species complex. Mycol Res 108:815–822. doi:10.1017/s0953756204000577. 15446715

[33] Nelson PE 1992 Taxonomy and biology of *Fusarium moniliforme*. Mycopathologia 117:29–36. doi:10.1007/bf00497276. 1513371

[34] Moretti A, Logrieco A, Bottalico A, Ritieni A, Fogliano V, Randazzo G 1996 Diversity in beauvericin and fusaproliferin production by different populations of *Gibberella fujikuroi* (*Fusarium* section *Liseola*). Sydowia 48:44–56.

[35] Leslie JF, Plattner RD, Desjardins AE, Klittich C 1992 Fumonisin B_1_ production by strains from different mating populations of *Gibberella fujikuroi* (*Fusarium* section *Liseola*). Phytopathology 82:341–345. doi:10.1094/Phyto-82-341.

[36] van Hove F, Waalwijk C, Logrieco A, Munaut F, Moretti A 2011 *Gibberella musae* (*Fusarium musae*) sp. nov.: a new species from banana closely related to *F. verticillioides*. Mycologia 103:570–585. doi:10.3852/10-038. 21177490

[37] Glenn AE, Zitomer NC, Zimeri AM, Williams LD, Riley RT, Proctor RH 2008 Transformation-mediated complementation of a *FUM* gene cluster deletion in *Fusarium verticillioides* restores both fumonisin production and pathogenicity on maize seedlings. Mol Plant Microbe Interact 21:87–97. doi:10.1094/MPMI-21-1-0087. 18052886

[38] Fotso J, Leslie JF, Smith JS 2002 Production of beauvericin, moniliformin, fusaproliferin, and fumonisins B_1_, B_2_, and B_3_ by fifteen ex-type strains of *Fusarium* species. Appl Environ Microbiol 68:5195–5197. doi:10.1128/AEM.68.10.5195-5197.2002. 12324376 PMC126425

[39] Moretti A, Mulè G, Ritieni A, Logrieco A 2007 Further data on the production of beauvericin, enniatins and fusaproliferin and toxicity to *Artemia salina* by *Fusarium* species of *Gibberella fujikuroi* species complex. Int J Food Microbiol 118:158–163. doi:10.1016/j.ijfoodmicro.2007.07.004. 17706820

[40] Santini A, Meca G, Uhlig S, Ritieni A 2012 Fusaproliferin, beauvericin and enniatins: occurrence in food–a review. World Mycotoxin J 5:71–81. doi:10.3920/WMJ2011.1331.

[41] Hamill RL, Higgens CE, Boaz HE, Gorman M 1969 Structure of beauvericin, a new depsipeptide antibiotic toxic to *Artemia salina*. Tetrahed Lett 10:4255–4258. doi:10.1016/S0040-4039(01)88668-8.

[42] Xu Y, Orozco R, Wijeratne EK, Gunatilaka AL, Stock SP, Molnár I 2008 Biosynthesis of the cyclooligomer depsipeptide beauvericin, a virulence factor of the entomopathogenic fungus *Beauveria bassiana*. Chem Biol 15:898–907. doi:10.1016/j.chembiol.2008.07.011. 18804027

[43] López-Berges MS, Hera C, Sulyok M, Schäfer K, Capilla J, Guarro J, Di Pietro A 2013 The velvet complex governs mycotoxin production and virulence of *Fusarium oxysporum* on plant and mammalian hosts. Mol Microbiol 87:49–65. doi:10.1111/mmi.12082. 23106229

[44] Zhang T, Zhuo Y, Jia XP, Liu JT, Gao H, Song FH, Liu M, Zhang L 2013 Cloning and characterization of the gene cluster required for beauvericin biosynthesis in *Fusarium proliferatum*. Sci China Life Sci 56:628–637. doi:10.1007/s11427-013-4505-1. 23832252

[45] Kuo CH, Ochman H 2010 The extinction dynamics of bacterial pseudogenes. PLoS Genet 6:e1001050. doi:10.1371/journal.pgen.1001050. 20700439 PMC2916853

[46] Balakirev ES, Ayala FJ 2003 Pseudogenes: are they “junk” or functional DNA? Annu Rev Genet 37:123–151. doi:10.1146/annurev.genet.37.040103.103949. 14616058

[47] Milligan MJ, Lipovich L 2015 Pseudogene-derived IncRNAs: emerging regulators of gene expression. Front Genet 5:476. doi:10.3389/fgene.2014.00476. 25699073 PMC4316772

[48] Liuzzi V, Mirabelli V, Cimmarusti M, Haidukowski M, Leslie JF, Logrieco A, Caliandro R, Fanelli F, Mulè G 2017 Enniatin and beauvericin biosynthesis in *Fusarium* species: production profiles and structural determinant prediction. Toxins 9:45. doi:10.3390/toxins9020045. PMC5331425 28125067

[49] Torres A, Reynoso MM, Rojo F, Ramírez ML, Chulze SN 2001 Fungal and mycotoxin contamination in home grown maize harvested in the north area of Argentina. Food Add Contaminants 18:836–843. doi:10.1080/02652030110046208. 11552751

[50] O'Donnell K, Cigelnik E 1997 Two divergent intragenomic rDNA ITS2 types within a monophyletic lineage of the fungus *Fusarium* are non-orthologous. Mol Phylogenet Evol 7:103–116. doi:10.1006/mpev.1996.0376. 9007025

[51] O'Donnell K, Kistler HC, Cigelnik E, Ploetz RC 1998 Multiple evolutionary origins of the fungus causing Panama disease of banana: concordant evidence from nuclear and mitochondrial gene genealogies. Proc Natl Acad Sci U S A 95:2044–2049. doi:10.1073/pnas.95.5.2044. 9482835 PMC19243

[52] Liu YL, Whelen S, Hall BD 1999 Phylogenetic relationships among ascomycetes, evidence from an RNA polymerase II subunit. Mol Biol Evol 16:1799–1808. doi:10.1093/oxfordjournals.molbev.a026092. 10605121

[53] Geiser DM, del Mar Jiménez-Gasco M, Kang S, Makalowska I, Veeraraghavan N, Ward TJ, Zhang N, Kuldau GA, O'Donnell K 2004 FUSARIUM-ID v. 1.0: a DNA sequence database for identifying *Fusarium*. Eur J Plant Pathol 110:473–479. doi:10.1023/B:EJPP.0000032386.75915.a0.

[54] Nguyen LT, Schmidt HA, von Haeseler A, Minh BQ 2015 IQ-TREE: a fast and effective stochastic algorithm for estimating maximum-likelihood phylogenies. Mol Biol Evol 32:268–274. doi:10.1093/molbev/msu300. 25371430 PMC4271533

[55] Tamura K, Nei M 1993 Estimation of the number of nucleotide substitutions in the control region of mitochondrial DNA in humans and chimpanzees. Mol Biol Evol 10:512–526. doi:10.1093/oxfordjournals.molbev.a040023. 8336541

[56] Felsenstein J 1985 Phylogenies and the comparative method. Am Nat 125:1–15. doi:10.1086/284325.

[57] Wingfield BD, Barnes I, Wilhelm de Beer Z, De Vos L, Duong TA, Kanzi AM, Naidoo K, Nguyen HDT, Santana QC, Sayari M, Seifert KA, Steenkamp ET, Trollip C, van der Merwe NA, van der Nest MA, Markus Wilken P, Wingfield MJ 2015 IMA genome-F5: draft genome sequences of *Ceratocystis eucalypticola, Chrysoporthe cubensis*, *C. deuterocubensis*, *Davidsoniella virescens*, *Fusarium temperatum*, *Graphilbum fragrans*, *Penicillium nordicum*, and *Thielaviopsis musarum*. IMA Fungus 6:493–506. doi:10.5598/imafungus.2015.06.02.13. 26734552 PMC4681265

[58] van der Nest MA, Beirn LA, Crouch JA, Demers JE, de Beer ZW, De Vos L, Gordon TR, Moncalvo J-M, Naidoo K, Sanchez-Ramirez S, Roodt D, Santana QC, Slinski SL, Stata M, Taerum SJ, Wilken PM, Wilson AM, Wingfield MJ, Wingfield BD 2014 Draft genomes of *Amanita jacksonii, Ceratocystis albifundus, Fusarium circinatum, Huntiella omanensis, Leptographium procerum, Rutstroemia sydowiana,* and *Sclerotinia echinophila*. IMA Fungus 5:472–486. doi:10.5598/imafungus.2014.05.02.11. PMC4329328 25734036

[59] Wiemann P, Sieber CMK, von Bargen KW, Studt L, Niehaus E-M, Espino JJ, Huß K, Michielse CB, Albermann S, Wagner D, Bergner SV, Connolly LR, Fischer A, Reuter G, Kleigrewe K, Bald T, Wingfield BD, Ophir R, Freeman S, Hippler M, Smith KM, Brown DW, Proctor RH, Münsterkötter M, Freitag M, Humpf H-U, Güldener U, Tudzynski B 2013 Deciphering the cryptic genome: genome-wide analyses of the rice pathogen *Fusarium fujikuroi* reveal complex regulation of secondary metabolism and novel metabolites. PLoS Pathog 9:e1003475. doi:10.1371/journal.ppat.1003475. 23825955 PMC3694855

[60] Wingfield BD, Ades PK, Al-Naemi FA, Beirn LA, Bihon W, Crouch JA, de Beer ZW, de Vos L, Duong TA, Fields CJ, Fourie G, Kanzi AM, Malapi-Wight M, Pethybridge SJ, Radwan O, Rendon G, Slippers B, Santana QC, Steenkamp ET, Taylor PWJ, Vaghefi N, van der Merwe NA, Veltri D, Wingfield MJ 2015 Draft genome sequences of *Chrysoporthe austroafricana*, *Diplodia scrobiculata*, *Fusarium nygamai*, *Leptographium lundbergii*, *Limonomyces culmigenus*, *Stagonosporopsis tanaceti*, and *Thielaviopsis punctulata*. IMA Fungus 6:233–248. doi:10.5598/imafungus.2015.06.01.15. 26203426 PMC4500086

[61] Ma LJ, van der Does HC, Borkovich KA, Coleman JJ, Daboussi MJ, Di Pietro A, Dufresne M, Freitag M, Grabherr M, Henrissat B, Houterman PM, Kang S, Shim W, Woloshuk C, Xie X, Xu J, Antoniw J, Baker S, Bluhm B, Breakspear A, Brown D, Butchko R, Chapman S, Coulson R, Coutinho P, Danchin E, Diener A, Gale L, Gardiner D, Goff S, Hammond-Kosack K, Hilburn K, Hua-Van A, Jonkers W, Kazan K, Kodira C, Koehrsen M, Kumar L, Lee Y, Li L, Manners J, Miranda-Saavedra D, Mukherjee M, Park G, Park J, Park S, Proctor R, Regev A, Ruiz-Roldan M, Sain D, Sakthikumar S, Sykes S, Schwartz D, Turgeon B, Wapinski I, Yoder O, Young S, Zeng Q, Zhou S, Galagan J, Cuomo C, Kistler H, Rep M 2010 Comparative genomics reveals mobile pathogenicity chromosomes in *Fusarium*. Nature 464:367–373. doi:10.1038/nature08850. 20237561 PMC3048781

[62] Brown DW, Butchko RA, Busman M, Proctor RH 2007 The *Fusarium verticillioides FUM* gene cluster encodes a Zn(II)_2_Cys_6_ protein that affects *FUM* gene expression and fumonisin production. Eukaryot Cell 6:1210–1218. doi:10.1128/EC.00400-06. 17483290 PMC1951116

[63] Stanke M, Schöffmann O, Morgenstern B, Waack S 2006 Gene prediction in eukaryotes with a generalized hidden Markov model that uses hints from external sources. BMC Bioinformatics 7:62. doi:10.1186/1471-2105-7-62. 16469098 PMC1409804

[64] Solovyev V, Kosarev P, Seledsov I, Vorobyev D 2006 Automatic annotation of eukaryotic genes, pseudogenes and promoters. Genome Biol 7(Suppl 1):S10–S12. doi:10.1186/gb-2006-7-s1-s10. 16925832 PMC1810547

[65] Darzentas N 2010 Circoletto: visualizing sequence similarity with Circos. Bioinformatics 26:2620–2621. doi:10.1093/bioinformatics/btq484. 20736339

[66] Frisvad JC, Smedsgaard J, Samson RA, Larsen TO, Thrane U 2007 Fumonisin B_2_ production by *Aspergillus niger*. J Agric Food Chem 55:9727–9732. doi:10.1021/jf0718906. 17929891

[67] de Girolamo A, Fauw DPD, Sizoo E, van Egmond H, Gambacorta L, Bouten K, Stroka J, Visconti A, Solfrizzo M 2010 Determination of fumonisins B_1_ and B_2_ in maize-based baby food products by HPLC with fluorimetric detection after immunoaffinity column clean-up. World Mycotoxin J 3:135–146. doi:10.3920/WMJ2010.1213.

[68] de Girolamo A, Lattanzio VM, Schena R, Visconti A, Pascale M 2014 Use of liquid chromatography–high-resolution mass spectrometry for isolation and characterization of hydrolyzed fumonisins and relevant analysis in maize-based products. J Mass Spectrom 49:297–305. doi:10.1002/jms.3342. 24719345

